# Role of the Backbenchers of the Renin-Angiotensin System ACE2 and AT2 Receptors in COVID-19: Lessons From SARS

**DOI:** 10.7759/cureus.8411

**Published:** 2020-06-02

**Authors:** Jyotsna Gumashta, Raghvendra Gumashta

**Affiliations:** 1 Physiology, All India Institute of Medical Sciences, Nagpur, IND; 2 Community Medicine, People's College of Medical Sciences and Research Centre, Bhopal, IND

**Keywords:** ace2, sars, covid-19, ras (renin angiotensin system), coronavirus, angiotensin receptor type 2 (at2r), angiotensin receptor type 1 (at1r), angiotensin ii (agii), ace (angiotensin-converting enzyme)

## Abstract

The novel coronaviruses causing severe acute respiratory syndrome (SARS) and coronavirus disease 2019 (COVID-19) have been shown to utilize angiotensin-converting enzyme 2 (ACE2) as the receptor for entry into the host cells. The involvement of the renin-angiotensin system (RAS) in the evolution and pathogenesis of lung diseases has been implicated in recent years. The two enzymes of RAS, angiotensin-converting enzyme (ACE) and ACE2, serve a contrasting function. ACE helps in the formation of angiotensin II (AGII) from angiotensin I (AGI), and ACE2 cleaves AGI and AGII into AG (1-9) and AG (1-7) respectively. The ACE-induced AGII has vasoconstrictor and pro-inflammatory properties via AT1R, whereas ACE2 has been shown to protect against lung injury. The less spoken about AGII receptor, angiotensin receptor type 2 (AT2R), has anti-inflammatory and anti-fibrotic effects in lung tissue and may be of significance in light of the lung pathology presentation in COVID-19. A review of articles searched in PubMed and peer-reviewed journals of importance was done using search terms “ACE2,” “AT2,” “SARS,” and COVID-19.” Lung involvement in both SARS and COVID-19 has been very severe and suggestive of severe inflammatory and immune reactions. Animal studies have shown that ACE2 and AT2 receptors counter the pro-inflammatory and other effects mediated by angiotensin II by their vasodilator, anti-inflammatory, anti-fibrotic, and anti-proliferative effects. They have been shown to protect against and revert acute lung injuries. The instrumental role of recombinant ACE2, AT2 receptor agonists, and AT1 receptor blockers may be helpful in the treatment of COVID-19.

## Introduction and background

The coronavirus 2019 (COVID-19) pandemic has affected over 3 million people and caused more than 2 lac deaths in over 200 countries over a duration of four months. There is no medicine or vaccine to date for treatment and symptomatic management is the present treatment plan. Severe acute respiratory syndrome (SARS) and Middle East respiratory syndrome (MERS) in the past and now COVID-19 are recurring epidemic threats to humans. This review has been undertaken to search probable modes that can be explored to find a drug treatment that can be used to treat COVID-19 or any similar illness involving mainly the respiratory system. The relatively benign coronavirus causing the common cold has taken a novel form, utilizing newer pathways to enter the host and causing severe disease in some cases. The coronaviruses contain a single-stranded 5’ capped positive-stranded ribonucleic acid (RNA) molecule that ranges from 26 to 32 kb. The viral membrane contains the membrane (M) glycoprotein, the spike (S) glycoprotein, the envelop (E) glycoprotein, and the nucleocapsid (N) protein. A member of the renin-angiotensin system (RAS), the angiotensin-converting enzyme 2 (ACE2) protein has been reported to be the entry-point receptor for severe acute respiratory syndrome coronavirus (SARS-CoV) and novel SARS-CoV2 causing COVID-19. The viral S protein interacts with the human ACE2, which is present as a transmembrane protein to gain entry into the host [1-3].

The two enzymes of the renin-angiotensin system (RAS), angiotensin-converting enzyme (ACE) and ACE2 serve contrasting functions. ACE converts angiotensin I (AGI) into angiotensin II (AGII) and AGII has vasoconstrictor and pro-inflammatory properties via its type1 receptor (AT1R). ACE inhibitors, e.g. lisinopril, and AT1R inhibitors, commonly known as angiotensin receptor blockers (ARB) like losartan, are very commonly used drugs for cardiovascular diseases like hypertension and cardiac failure. The involvement of RAS in the evolution and pathogenesis of lung diseases has received attention in recent years [4].

The histopathological reports of post-mortem studies of COVID-19 deaths show diffuse alveolar damage, hyaline membrane deposition, fibrin exudates, and consolidation by fibroblastic proliferation with the extracellular matrix and fibrin-forming clusters in air spaces [5-6]. Post-mortem findings of SARS deaths showed features of diffuse alveolar damage (DAD) with pronounced pulmonary edema and hyaline membrane formation. In some areas, there was interstitial thickening, with mild to moderate fibrosis. [7-8] The ACE homolog ACE2, which is the entry receptor for the virus, is expressed abundantly in type II alveolar cells and may have been a base for the rapid expansion of SARS-CoV and a vicious circle of local alveolar wall destruction, resulting in rapidly progressive severe diffuse alveolar damage [9]. Considering the post-mortem findings of the lung, the same may be true in COVID-19. As opposed to ACE, soluble ACE 2 has been shown to protect against lung injury. The less spoken about angiotensin II receptor type 2 (AT2R) has anti-inflammatory and anti-fibrotic effects in lung tissue and may be of significance in light of the lung pathology presentation in COVID-19. It is imperative to assess the role of ACE2 and AT2R as the natural but forgotten protectors of the alveolar environment, along with ARBs, in severe acute lung disease by the novel coronavirus.

## Review

Angiotensin-converting enzyme (ACE) and its homolog (ACE2)

Angiotensin-converting enzyme (ACE) is a dipeptidyl carboxypeptidase expressed predominantly in the lung capillary endothelium, and it converts the decapeptide angiotensin I (AGI) to the octapeptide angiotensin II (AGII). AGII has two main receptors, AT1R and AT2R. Angiotensin II via its AT1R receptor causes vasoconstriction along with many other effects on the kidneys and brain to regulate the body fluid volume and blood pressure. It also has pro-inflammatory properties. A study suggested that the activation of the pulmonary RAS influences the pathogenesis of acute lung injury, acute respiratory distress syndrome, and SARS, as they are attenuated by blocking the renin-angiotensin pathway [10]. Thus, the inhibition of the ACE/AGII/ATR1 axis may be beneficial in the setting of its enhanced activity. Both ACE inhibitors and ARBs are used clinically to block the effects of AGII but the ARBs may have a much greater potential to block the renin-angiotensin system than ACE inhibition because an estimated 40% of AG II is formed via non-ACE pathways (e.g. chymase) in humans [11-12].

In the year 2000, a new homolog of ACE was identified separately by two groups and was named ACE2 [13-14]. ACE2 is a zinc metalloprotease with a 40% identity and a 61% similarity to ACE [13]. ACE2 converts AGI to AG (1-9) and AGII to AG (1-7) by removing one amino acid from each. ACE2 is not inhibited by classical ACE inhibitors like captopril, enalapril, and lisinopril [14]. Both the authors reported that ACE2 is expressed mainly in the heart, kidney, and testis [13-14]. Later, in a study using immunolocalization of the ACE2 protein, it was found that ACE2 is expressed in many tissues, including type I and type II alveolar pneumocytes, nasal, oral and nasopharyngeal mucosa, enterocytes of the small intestine, endothelial cells and smooth muscles of blood vessels of all the tissues studied, and weakly in bronchial epithelial cells [9].

Role of ACE2 in virus entry and lung protection

Numerous animal studies have been conducted to study ACE2 after the discovery that SARS-CoV used it as a portal for entry. These studies may be projected to COVID-19, as its virus also gains entry through the same receptor. The picture in severe cases in both SARS and COVID-19 is that of acute respiratory distress. It was demonstrated in mice that ACE2 was required for the effective replication of SARS-CoV in vivo as ACE2 knockout mice showed a very low quantity of virus when they were infected with SARS-CoV [15]. On one hand, where ACE2 was proved to be the entry receptor for novel CoV, on the other hand, ACE2 knockout mice showed very severe acute lung injury in aspiration, endotoxin, and peritoneal sepsis-induced acute respiratory distress syndrome (ARDS) models. Loss of ACE2 expression in mutant mice resulted in enhanced vascular permeability, increased lung edema, neutrophil accumulation, and worsened lung function [10]. Thus, ACE2 protects murine lungs from ARDS in ACE2 knockout mice. This protective effect of ACE2 is probably by reducing AGII by breaking it to AG (1-7) [10]. Hence, there is a protective effect of ACE2 in acute lung injury with AGII being the probable target. In another study on mice, downregulation of ACE2 expression by SARS-CoV infections and its spike protein was reported. A study also reported that treatment with exogenous recombinant ACE2 protein improved symptoms of acute lung injury in wild type mice as well as ACE2 knockout mice [16]. Thus, ACE2 has a dual role, one, to support virus entry into the host and, two, to protect against severe lung injury.

In relation to ACE2, the findings of studies comparing viruses NL63 and SARS-CoV are intriguing. NL63 is another human coronavirus that uses the same ACE2 for entry as does SARS-CoV but causes mild respiratory disease. Its interaction with ACE2 is weaker than the SARS-CoV S protein interaction with ACE2. NL63-CoV and SARS-CoV have no structural homology in the receptor-binding domain (RBD), yet the two viruses recognize common ACE2 regions [17]. Whether it is lower-affinity interaction with ACE-2 or the difference in ACE-2 signaling following SARS-CoV and NL63 S protein binding or it is the S protein itself that accounts for the differential pathogenicity of human coronavirus NL63 and SARS-CoV remains to be determined [17-18]. Recently, it has been demonstrated that the COVID-19 CoV binds with ACE2 as avidly as SARS-CoV [19]. In a study comparing the two viruses, it was reported that the SARS-CoV binds more efficiently to ACE2 than NL63 and correlates with ACE2 shedding [20]. But working ahead on these findings, with a revised experimental setup, another study found that the ACE2 shedding with NL63 was robust and depended on the fold increase in viral replication and occurred during the early phase of replication [21]. With these observations, it appears that the severity of the disease depends on the efficiency of the binding of the spike protein with ACE2, as both SARS-CoV and CoV2 have greater affinity than NL63. Secondly, the shedding and downregulation of ACE2 expression does not seem to determine the potential severity of lung disease but is associated with higher viral replication. A study in 2004 found that the soluble ACE2 ectodomain specifically blocked infection by SARS-CoV S-bearing pseudotypes, and this finding may be a breakthrough for COVID-19 also [22]. Thus, recombinant ACE2 may be beneficial in blocking virus entry and in reducing lung injury in COVID-19 via some missing link. Thus the double-edged sword ACE2 must be explored to be used appropriately for conquering COVID-19 by strategic administration through its considered inclusion in the predefined and agreed-on treatment protocol of COVID-19.

Angiotensin II receptor subtype (AT2R)

AGII, the major effector peptide of the RAS, promotes vasoconstriction, proliferation, inflammation, and fibrosis within the pulmonary vasculature and lung parenchyma via stimulation of AT1 receptors. The use of losartan to block the AT1 receptor improved lung injury in this mouse model. The aforementioned detrimental effects of AGII may be counterbalanced by the activation of AT2 receptors [23].

AT2R has not been given much attention after its discovery, as most of the functions of AGII were operated via AT1R and the significance of AT1R in cardiovascular and renal pathologies became well-known. The expression of AT2R in healthy adults is often low and is mainly identified in the renal, cardiovascular, adrenal medulla, brain tissues, myometrium, and ovaries [24-25] In contrast, in fetal tissues, AT2R is the dominant receptor subtype [26]. In the adult, AT2Rs are re-expressed or upregulated under certain pathophysiological conditions, such as mechanical injury or ischemia like myocardial infarction, vascular injury, brain ischemia, and renal failure [24,27]. AT2R exerts vasodilator, anti-fibrotic, and anti-inflammatory effects in a variety of disease models, as well as natriuretic and antihypertensive effects in renal disease [28]. The AT2R-specific agonist may effectively dampen the pro-inflammatory and aggressive behavior in rheumatoid synovitis [29]. Thus, AT2R has proven anti-inflammatory effects in a variety of tissues. The activation of AT2R is thought to counter-regulate the pathophysiological effects of angiotensin II (AGII) induced by AT1R [24].

AT2R research gained momentum when the first nonpeptide AT2R agonist, compound 21 (C-21) was introduced in 2004 [24]. A study investigated the effect of the AT2R agonist C-21 in the bleomycin model of pulmonary fibrosis and reported the beneficial effects of C-21, which were associated with decreased infiltration of macrophages in the lungs, reduced lung inflammation, and diminished pulmonary collagen accumulation [30]. In the monocrotaline (MCT)-induced pulmonary hypertension rat model, C-21 treatment reversed both interstitial and perivascular fibrosis. Furthermore, a decrease in ACE2 messenger RNA (mRNA) levels was observed in MCT-induced pulmonary hypertension animals, which was reversed by C-21 therapy with a two-fold increase in ACE2 levels and a concomitant decrease in ACE expression [23]. The protective effect of ACE2 has already been emphasized. Furthermore, C-21 treatment normalized the AT1/AT2 receptor ratio to restore the lung RAS balance. C-21 treatment prevented, as well as attenuated, the progression of lung fibrosis and the accompanying pulmonary hypertension. Thus, in view of the above inferences, the acute inflammatory lung pathology being observed in COVID-19 may be ameliorated by the AT2R agonist.

## Conclusions

Three classes of drugs affecting RAS may be candidates to ameliorate the acute lung pathology in COVID-19. With the possibility of AGII overexpression as the trigger in COVID-19 lung pathology, its inhibition may be of benefit. Here, ARBs like losartan may be more beneficial than ACE inhibitors for the fact that sufficient AGII is formed by ACE non-dependent mechanisms. CoV2 uses ACE2 to gain entry into the host, and after gaining entry, it causes the shedding and downregulation of ACE2 to sabotage its anti-inflammatory and protective effect. This, along with the observation that the soluble ACE2 ectodomain specifically blocked infection by SARS-CoV S-bearing pseudotypes, shows that the use of soluble recombinant ACE2 may be helpful in reducing lung injury in COVID-19. Our speculation is that AT2R may be re-expressed in COVID-related lung injury and hence the use of AT2R agonists may also be explored to elicit their anti-inflammatory and anti-fibrotic effects. Bacterial communicable diseases were a menace and created havoc prior to the antibiotic era. However, during the last few decades, public health focus has largely been on non-communicable diseases and related lifestyle interventions. The sudden spurt of COVID-19 and its global spread demands innovative approaches using forgotten enzymatic pathways, as suggested in this study, for magnanimous improvements in the clinical outcome among the COVID-19 cases. The herein suggested interventions with ACE2 and AT2 receptors agonist, along with ARBs, may be beneficial in improving the clinical outcome of COVID-19.
